# Histology of the Ovary of the Laying Hen (*Gallus domesticus*)

**DOI:** 10.3390/vetsci4040066

**Published:** 2017-12-11

**Authors:** K. Denise Apperson, Karyn E. Bird, Gita Cherian, Christiane V. Löhr

**Affiliations:** 1 College of Veterinary Medicine, Oregon State University, Corvallis, OR 97331, USA; appersonkd@gmail.com (K.D.A.), karyn.bird@oregonstate.edu (K.E.B.); 2 Department of Animal and Rangeland Sciences, Oregon State University, Corvallis, OR 97331, USA; gita.cherian@oregonstate.edu

**Keywords:** laying hen, epithelial ovarian cancer, histology, rete ovarii, epoöphoron, ovarian surface epithelium

## Abstract

The laying hen (*Gallus domesticus*) is a robust animal model for epithelial ovarian cancer. The use of animal models is critical in identifying early disease markers and developing and testing chemotherapies. We describe the microscopic characteristics of the normally functioning laying hen ovary and proximal oviduct to establish baselines from which lesions associated with ovarian cancer can be more readily identified. Ovaries and oviducts were collected from 18-month-old laying hens (*n* = 18) and fixed in 10% neutral buffered formalin. Hematoxylin- and eosin-stained sections were examined by light microscopy. Both post-ovulatory follicular regression and atresia of small follicles produce remnant clusters of vacuolated cells with no histological evidence that scar tissue persists. Infiltrates of heterophils are associated with atresia of small follicles, a relationship not previously documented in laying hen ovaries. Because these tissues can be mistaken for cancerous lesions, we present a detailed histological description of remnant Wolffian tissues in the laying hen ovary. Immunohistochemical staining for pancytokeratin produced a positive response in ovarian surface epithelium and staining for vimentin produced a positive response in granulosa cells of follicles. Epithelial cells lining glands of the remnant epoöphoron had a positive response to both pancytokeratin and vimentin, a result also observed in women.

## 1. Introduction

Epithelial ovarian cancer (EOC) occurs in the surface epithelium of ovaries of women. Women with EOC have a lower relative five-year survival rate than women with other gynecological cancers [1] despite a similar incidence rate for those cancers [2]. The low survival rate for EOC is primarily associated with diagnosis late in the progression of the disease, often after it has metastasized to other organs [2]. There are few specific symptoms associated with early stages of the disease and little information is available on early expression and progression of EOC in women [3]. Early detection is a significant issue in devising improved treatment therapies.

The laying hen (*Gallus domesticus*) is a robust animal model for epithelial ovarian cancer [3,4,5,6,7,8]. Laying hens spontaneously develop EOC [3,5,6,9,10,11,12] with an incidence as high as 24% in hens older than two years [13]. Epithelial ovarian cancer in other animal models is usually induced experimentally or by genetic engineering [8,11]. In hens, EOC has a gross and microscopic morphology, histopathology, and course of development that are similar to the disease in women [4,12,14,15]. In women and laying hens, EOC tumors express many of the same genes [11,15,16].

In both hens and women, the risk of developing EOC increases with increasing age and the number of lifetime ovulations. By the time laying hens reach two years of age, they have typically produced over 500 eggs, about the same number of ovulations experienced by a woman who has reached menopause. The “incessant ovulation” theory proposes that multiple cycles of tissue damage and repair, accumulation of unrepaired DNA damage, and constant exposure to inflammatory cytokines and proteinases initiate and promote carcinogenesis in the ovarian surface epithelium [2,9,10,17,18,19].

Epithelial cells in EOC tumors are not normal constituents of the ovary [20]. Tumors of EOC in women are classified into histological subtypes based on the similarity of cells in the tumors to different types of Müllerian (mesonephric) epithelia [12,20]. High-grade serous carcinomas comprise around 80% of EOC tumors in women [17,21]. Cells in serous tumors resemble ciliated epithelial cells of the proximal oviduct and infundibulum. An oviductal origin for this particular histological tumor subtype is not incompatible with the incessant ovulation theory [20,21]. The juxtaposition of the infundibulum of the oviduct and the ovary in both women and laying hens provides an easy route for malignant cell migration from oviductal epithelium to damaged OSE.

A thorough literature search did not yield a detailed description of the microscopic characteristics of the normally functioning laying hen ovary and proximal oviduct in either the veterinary medical literature or the ovarian cancer research literature. In this paper, we address this knowledge gap. Our objective is to establish a baseline from which lesions associated with pre-cancerous structural changes of the ovarian surface epithelium can be more readily identified in laying hen ovaries. In addition, establishing a baseline for the histological appearance of the proximal oviduct in laying hens that have not yet developed detectable tumors in either the oviduct or the ovarian surface epithelium may make identification of pre-neoplastic changes in oviductal tissue more consistent.

## 2. Materials and Methods

At 18 weeks of age, 18 brown Leghorn laying hens were randomly placed in individual wire cages (46 cm × 53 cm × 58 cm). The birds were provided with standard layer diets (16% crude protein, 2700 kcal/kg ME) and water ad libitum. The hens comprised the control group in a nutritional study. Conditions of 17 h of light and 7 h of darkness were maintained throughout the study. Reproductive tissues were collected as byproducts at the conclusion of the original nutritional study. Each bird was considered to be an experimental unit. Egg production for each bird was recorded daily. Birds were housed and handled in accordance with Oregon State University Institutional Animal Care and Use Committee protocols (ACUP #4713, approved July 2015).

At 18 months of age, the birds were weighed (median live body weight, 4.7 kg) and then euthanized with CO_2_ gas. Ovaries and oviducts were grossly examined then collected and fixed in 10% neutral buffered formalin. Formalin-fixed tissues were routinely processed and embedded in paraffin (Oregon Veterinary Diagnostic Laboratory, Corvallis, OR, USA). Hematoxylin- and eosin-stained (H & E), 3–5 micrometer sections were examined by light microscopy for microscopic characteristics. A trichrome stain was applied to select sections to highlight key features (Sigma-Aldrich Accustain Trichrome Stains (Masson); Procedure No. HT 15; St. Louis, MO, USA).

Immunohistochemistry (IHC) for pancytokeratin and vimentin was performed on an autostainer (Dako Autostainer Universal Staining System; Dako, Carpinteria, CA, USA) according to Oregon Veterinary Diagnostic Laboratory standard operating procedures. Paraffin sections were deparaffinized, and endogenous peroxidase activity was blocked by immersing slides in methanol containing 3% hydrogen peroxide for 10 min. Either a primary rabbit antiserum against bovine muzzle epidermal keratin subunits with wide-spectrum screening (WSS, Z0622, Dako Cytomation, Carpinteria, CA, USA; 1:500) or a monoclonal mouse antibody against human vimentin (Vim 3B4, Dako Cyomation, Carpenteria, CA, USA; 1:500) was applied for 30 min at room temperature. MaxPoly-One Polymer HRP Rabbit or HRP Mouse Detection solution (MaxVision Biosciences, Bothell, WA, USA) was applied for 7 min at room temperature followed by Nova Red (SK-4800; Vector Labs, Burlingame, CA, USA) as chromagen and Dako hematoxylin (S3302) as counterstain. Serial sections of chicken ovaries incubated with Dako Universal negative serum served as negative controls. Serial sections of normal canine skin were used as positive method controls.

## 3. Results

### 3.1. Overview of Gross and Microscopic Anatomy and Histology of the Laying Hen Ovary

**Figure 1:**
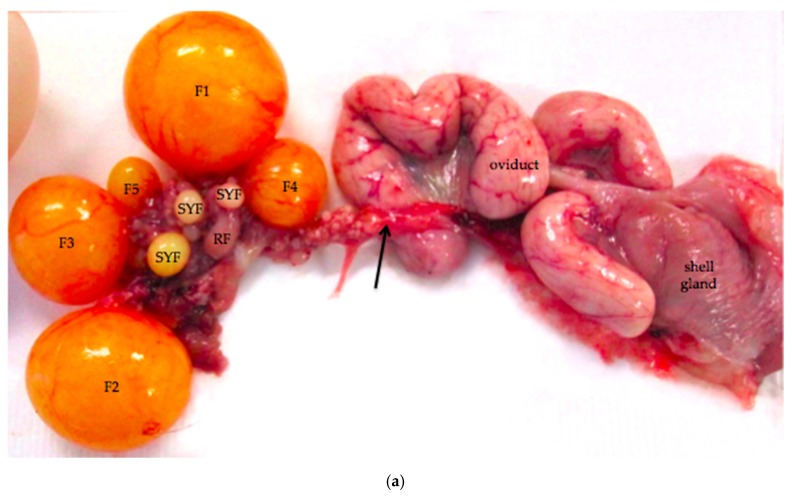

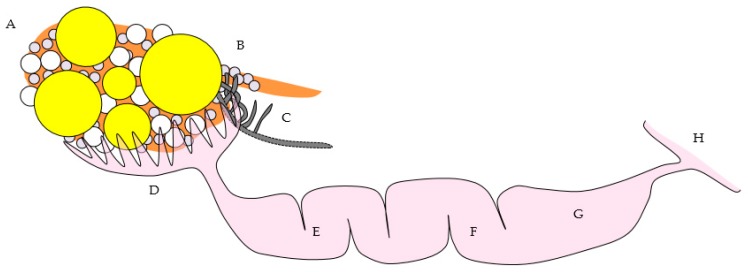
(**a**) Reproductive tract from an 18-month old laying hen. Five pre-ovulatory follicles are present (F1–F5). Small yellow follicles (SYF) and a regressing post-ovulatory follicle (RF) are visible. The arrow marks nerves and blood vessels surrounded by mesothelium that enter the ovary at the ovarian hilus. The oviduct and shell gland are labeled; (**b**) Schematic drawing of the laying hen reproductive tract showing the spatial relationship of the main anatomic elements. A Ovary with large pre-ovulatory follicles (yellow), small yellow follicles (white), small white follicles (grey); B hilus region of ovary where nerves and vessels enter the ovarian medulla; C Wolffian remnant tissues (rete ovarii in the medulla, extraovarian rete tubules and epoöphoron in the mesosalpinx or mesovarium); D infundibulum of the oviduct; E magnum of the oviduct; F isthmus of the oviduct; G shell gland; H cloaca.

**Figure 2:**
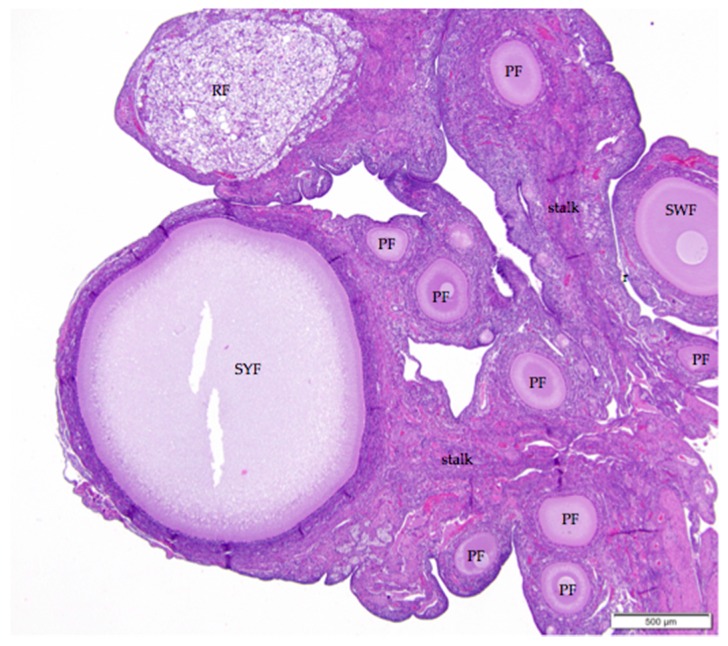
The laying hen ovary has a complex three-dimensional topography. The more eosinophilic medulla of the mature laying hen ovary can usually be distinguished from the more basophilic cortex containing the follicles. Larger follicles, such as the small yellow follicle (SYF) in this image, extend from the surface on stalks (labeled) with cores of connective, nervous, and vascular tissue. Follicles in all states of development can be observed in ovaries of reproductively active birds. This image also includes small white (SWF), primary (PF), primordial (unlabeled), and regressing (RF) follicles. H & E. 40×.

The ovarian cortex of reproductively active birds simultaneously contains a hierarchy of follicles in all stages of development from primordial follicles to large, yellow, yolk-filled pre-ovulatory follicles (Figure 1). Large follicles protrude from the surface of the ovary on short stalks while small follicles bulge from the surface (Figure 2). A section through avian ovarian tissue will intersect this topography at many different angles, complicating histological interpretations.

**Figure 3:**
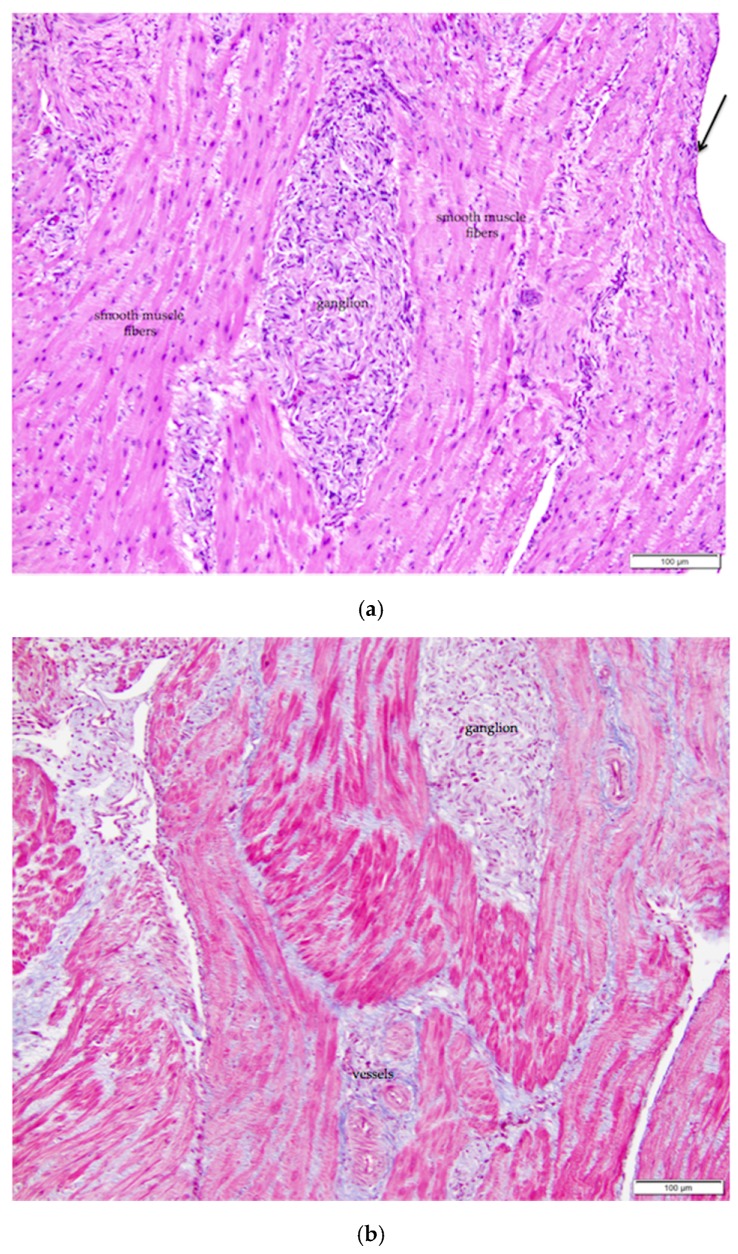
(**a**) The ovarian medulla of the mature laying hen consists of smooth muscle, collagenous connective tissue, ganglia, and large vessels. The arrow in the upper right of the image indicates simple squamous ovarian surface epithelium. H & E. 200×; (**b**) Section overlaps with that in (**a**). Masson’s trichrome stain has been applied. With this stain, smooth muscle fibers of the ovarian medulla and the arterial media are stained red and collagen tissue is stained blue 200×.

The mature ovarian cortex and medulla are intermingled, but they can usually be distinguished in ovaries of actively egg-laying hens. Cells of the cortex are more basophilic, while the medulla is more eosinophilic (Figure 2). The ovarian medulla consists of elongated, variably oriented bands of smooth muscle interspersed with regions of collagenous connective tissue and numerous fibroblasts (Figure 3a). Masson’s trichrome stain applied to the tissue highlights the alternating bands of smooth muscle and collagen (Figure 3b). Medullary vessels are larger than those in the cortex, and arteries can be tortuous. Small clusters of lymphocytes and occasional macrophages are scattered throughout the medulla.

Most avian species retain only the left ovary, oviduct, and shell gland at hatch. Falcons, eagles, and vultures are notable exceptions [22]. The left ovary is located in the coelomic cavity adjacent to the rostral end of the left kidney. The left adrenal gland and left ovary can share a common connective tissue capsule [23,24]. We observed that the adrenal gland is typically embedded in adipose tissue in the region of the ovarian hilus and pieces of this gland and its associated nerves and vessels are often collected along with ovarian tissue.

**Figure 4:**
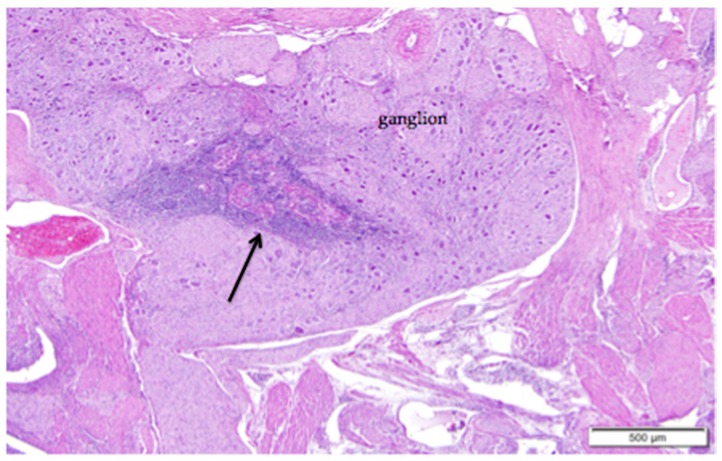
Ganglia in the region of the ovarian hilus can be associated with either the ovary or the left adrenal gland. Infiltrates of lymphocytes that cluster around vessels in the perineurium (arrow) are a common finding. Eosinophilic tissue of the ovarian medulla surrounds the neural tissue. H & E. 40×.

Large vessels and nerves enter the ovary at the ovarian hilus (Figure 1). The left cranial renal artery is usually the main blood supply to the ovary. Two large ovarian veins drain into the caudal vena cava. Infiltrates of leukocytes are located in the perineurium of ovarian and adrenal ganglia (Figure 4).

### 3.2. Ovarian Surface Epithelium

**Figure 5:**
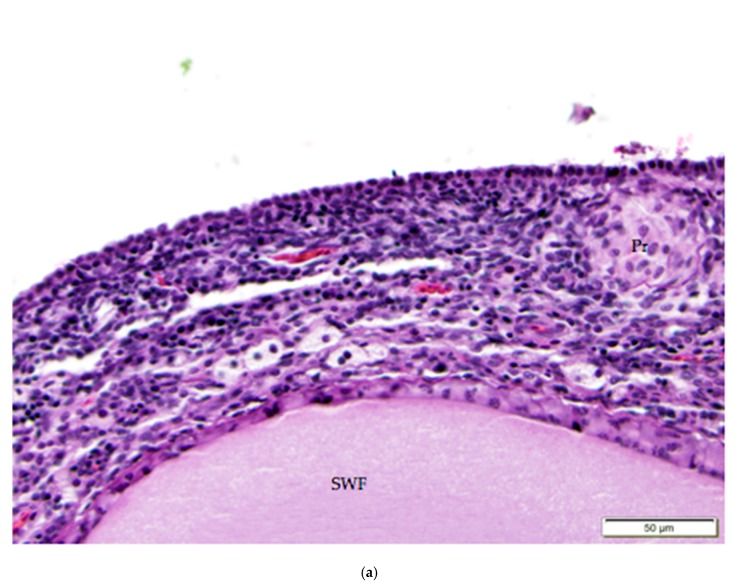

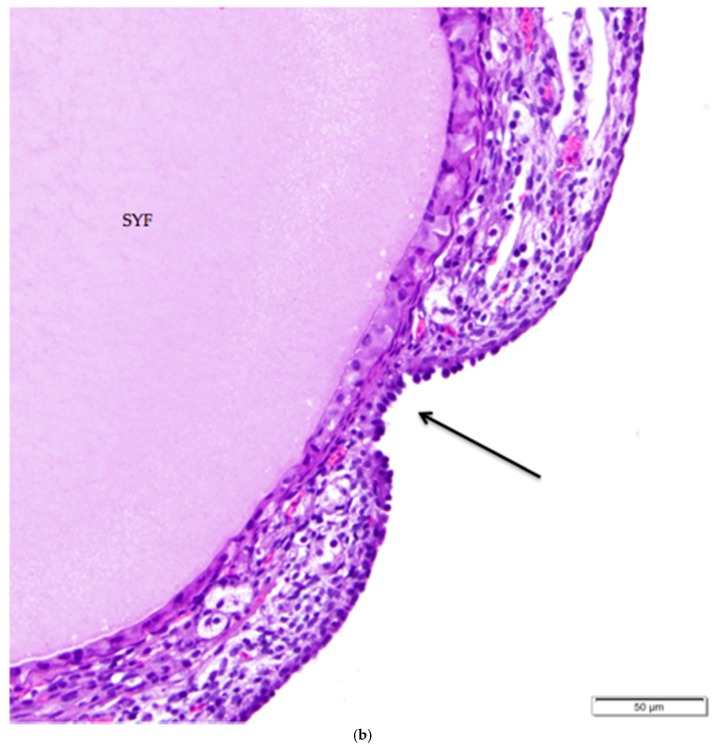
(**a**) Cuboidal ovarian surface epithelium adjacent to a small white follicle (SWF) and a primordial follicle (Pr). H & E 400×; (**b**) Simple squamous and cuboidal ovarian surface epithelium overlying a small yellow follicle (SYF). The arrow indicates a section of more proliferative, low columnar surface epithelium located at the follicular stoma along which ovulation would have occurred for this follicle. H & E. 400×.

The tunica albuginea, a thin layer of dense connective tissue, comprises the capsule of the ovary. Overlying the tunica albuginea is a serous membrane covered with a single layer of modified mesothelial cells called the ovarian surface epithelium (OSE). In laying hens, the ovarian surface epithelium may appear as simple squamous, simple cuboidal, or low columnar (Figure 5). Lateral cell boundaries between these epithelial cells are not distinct. Nuclei are darkly basophilic, round to oval, and centrally located. Nuclei of squamous epithelial cells are flattened. The relatively scanty cytoplasm is also basophilic. We observed that the state of development of adjacent follicles has some effect on the appearance of the surface epithelium, but this is not the only factor. For example, we observed that the ovarian surface epithelium often has a cuboidal appearance where it overlies small follicles (Figure 5a). Large follicles that protrude from the surface of the ovary are typically covered by simple squamous surface epithelium. The follicular stoma, an avascular line along which a follicle will ovulate, is characterized by more proliferative, low columnar surface epithelium (Figure 5b). When medullary tissue is directly overlain by the surface epithelium with no intervening cortical stroma, the surface epithelium also has a simple squamous appearance (Figure 3a).

**Figure 6:**
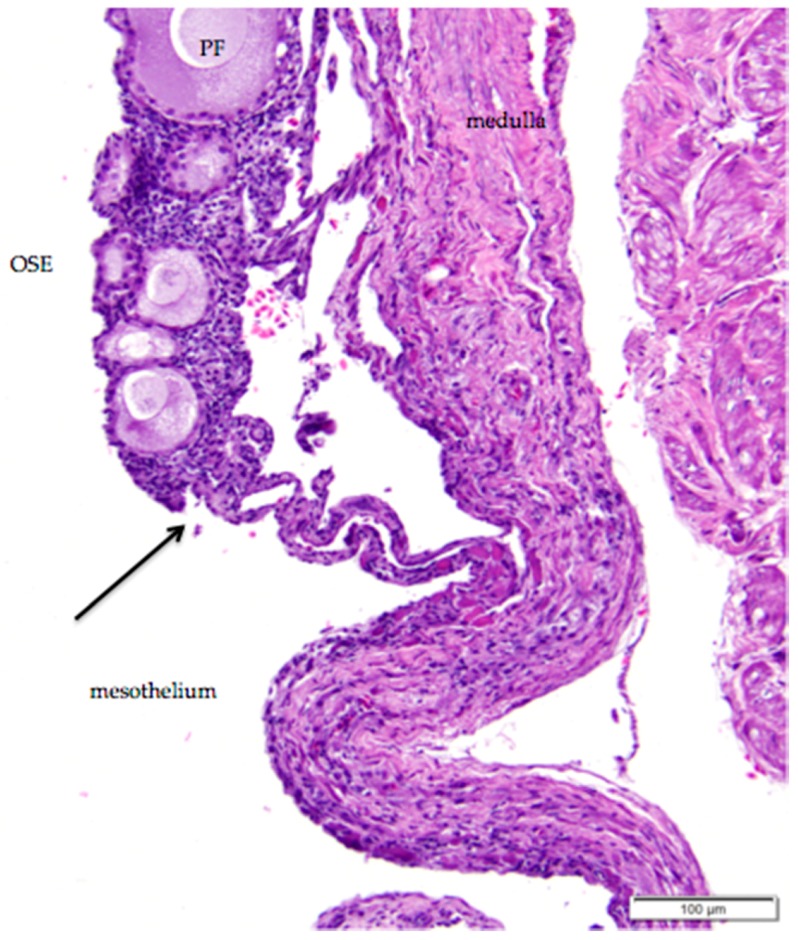
The simple cuboidal ovarian surface epithelium (OSE) overlying primordial follicles transitions in the ovarian hilus region to simple squamous mesothelium. There is a loss of follicles and ovarian cortical stromal cells at the transition (arrow). A primary follicle is labeled (PF); other follicles in the image are primordial follicles. H & E. 200×.

The histology of the surface epithelium in the region of the ovarian hilus of the laying hen has not previously been described in detail. There is generally an increase in the number of primordial follicles in the region of the ovarian hilus. We observed that simple cuboidal ovarian surface epithelium overlying these follicles transitions to simple squamous mesothelium at the point at which small follicles and cortical stroma are no longer present (Figure 6). The squamous mesothelium in the hilar region is continuous with the mesothelial lining of the coelomic cavity.

### 3.3. Follicles and Endocrine Cells in the Ovarian Cortex

**Figure 7:**
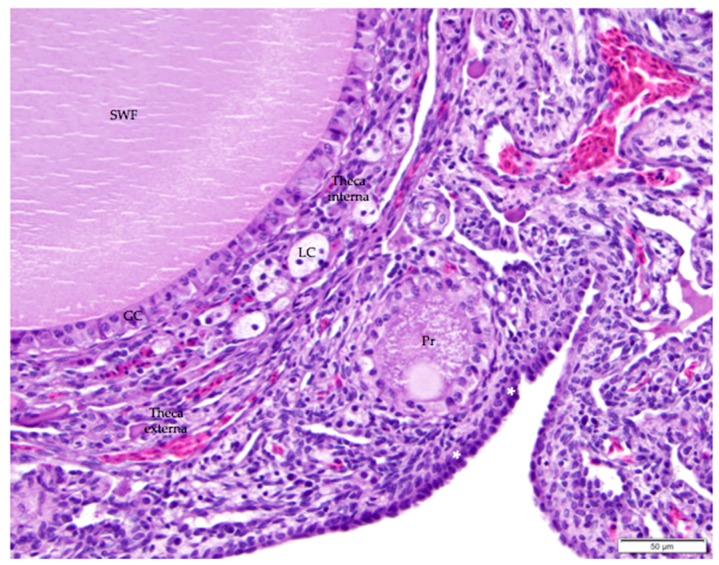
The thin, collagenous tunica albuginea that separates the surface epithelium from the ovarian cortex is occasionally visible (white asterisks). The image includes a small white follicle (SWF) and a primordial follicle (Pr). A single layer of granulosa cells (GC) delineates each follicle. The small white follicle is also surrounded by theca interna and theca externa layers. Luteal cell clusters (LC) are visible in the theca interna layer. H & E. 400×.

Follicles are delineated by a single layer of granulosa cells surrounding each ovum. Granulosa cells are large cuboidal cells with large, round to oblong heterochromatic nuclei that are positioned at the base of the cell (Figure 7). In more developed follicles, granulosa cells range in appearance from columnar to pseudostratified columnar to polyhedral. Cytoplasm is palely basophilic to amphophilic and can be granular, particularly in granulosa cells surrounding more developed follicles since these cells have a role in transporting yolk into the follicle [24,25]. Cell walls between adjacent granulosa cells are usually distinct.

Luteal cells are a type of stromal endocrine cell that typically occur in small clusters. The cells are large with clear, finely vacuolated cytoplasm (Figure 7). Nuclei are small, dark, and dense, and typically centered in the cell. Boundaries between luteal cells in clusters are indistinct. Luteal cell clusters are present throughout the ovarian cortex, but small clusters are always distributed in the thecal layers surrounding follicles (Figure 7). For this reason, some sources refer to them as “thecal glands” [26,27].

When primary follicles develop into small white follicles, stromal cells in the ovarian cortex surrounding the follicle differentiate into theca interna and theca externa layers (Figure 7). An eosinophilic, collagenous basement membrane separates granulosa cells from cells in the thecal layers. Granulosa and thecal layers may not be distinct in all small white follicles. Thecal cell nuclei are small, dark, and often elongated. Thecal cells have scant cytoplasm and indistinct lateral connections. The theca interna layer comprises fibroblasts and interstitial endocrine cells, including luteal cell clusters, as well as flattened thecal cells. Collagen fibers are diffusely distributed. Nerves and vessels propagate in the theca interna layer. The theca externa layer comprises similar types of cells and is mainly distinguished by its lack of luteal cell clusters. Little to no cortex may separate the theca externa layer and the surface epithelium once a developing follicle begins to protrude from the surface of the ovary (Figure 2).

Other stromal cells in the ovarian cortex are not associated with individual follicles. These cells are large, round to polyhedral with pale basophilic cytoplasm and large, elliptical, pale nuclei with clumped chromatin. Cell boundaries between adjacent cells are indistinct.

### 3.4. Follicular Regression and Atresia

**Figure 8:**
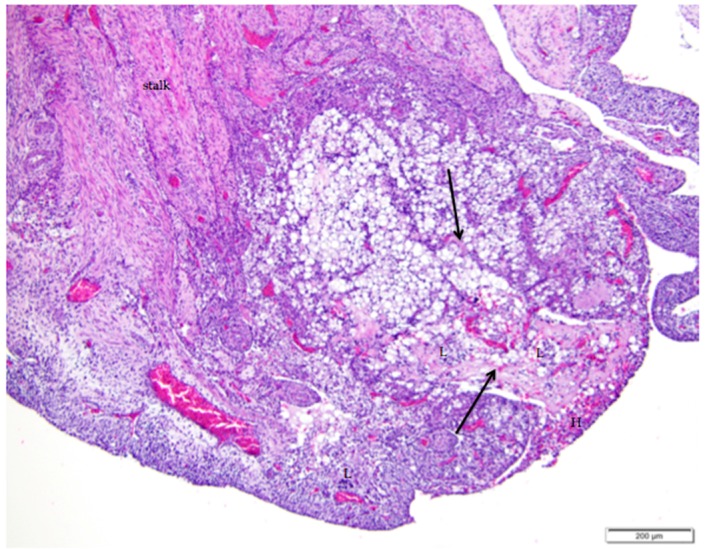
Regression of the post-ovulatory follicle occurs as orderly apoptosis in granulosa and thecal cells immediately after ovulation. Cords of connective tissue and fibroblasts (arrows) extend from the stalk (labeled) into the follicle as the basement membrane beneath the granulosa cell layer folds and creases into the follicular space. Vacuolar cells form as granulosa and thecal cells become filled with lipid inclusions. Infiltrates of heterophils (H) and lymphocytes (L) are distributed within the regressing follicle. H & E. 100×.

The actively reproducing laying hen maintains five or six large, yolk-filled follicles comprising a pre-ovulatory hierarchy. Following ovulation, the follicle undergoes regression. Granulosa and thecal cells swell and become vacuolated as they fill with lipid inclusions, detach from their neighbors, and develop peripherally located, pyknotic nuclei (Figure 8). These vacuolated cells resemble luteal cells, but the eccentric nucleus is characteristic of vacuolated cells in the regressing follicle.

**Figure 9:**
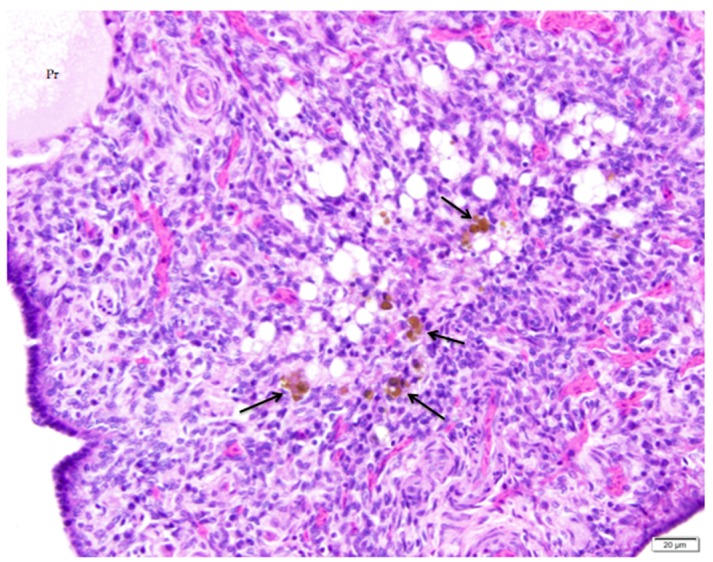
A nearly-completely regressed, post-ovulatory follicle is indicated by the remnant of vacuolated cells with pyknotic nuclei. Macrophages containing golden-brown hemosiderin are commonly observed (arrows). An adjacent primordial follicle is labeled (Pr). H & E. 500×.

Cords of connective tissue, fibroblasts, and small vessels form in the collapsed follicle as the basement membrane underlying the granulosa cells folds inward, drawing cells in the thecal layers with it (Figure 8). As the post-ovulatory follicle regresses by orderly apoptosis, smooth muscle and connective tissue that extend from the ovarian medulla to form the core of the stalk typically enfold the mass of cells in a semispherical cup-shaped structure. We found no histological evidence that this increase in fibrous or collagenous tissue persists in the cortex in the region of fully regressed follicles. We observed infiltrates of lymphocytes and heterophils in and around regressing follicles (Figure 8). Regression continues until only a small remnant of vacuolated cells is present in the cortex. Macrophages containing golden-brown hemosiderin are commonly observed among remnants of vacuolated cells (Figure 9).

**Figure 10:**
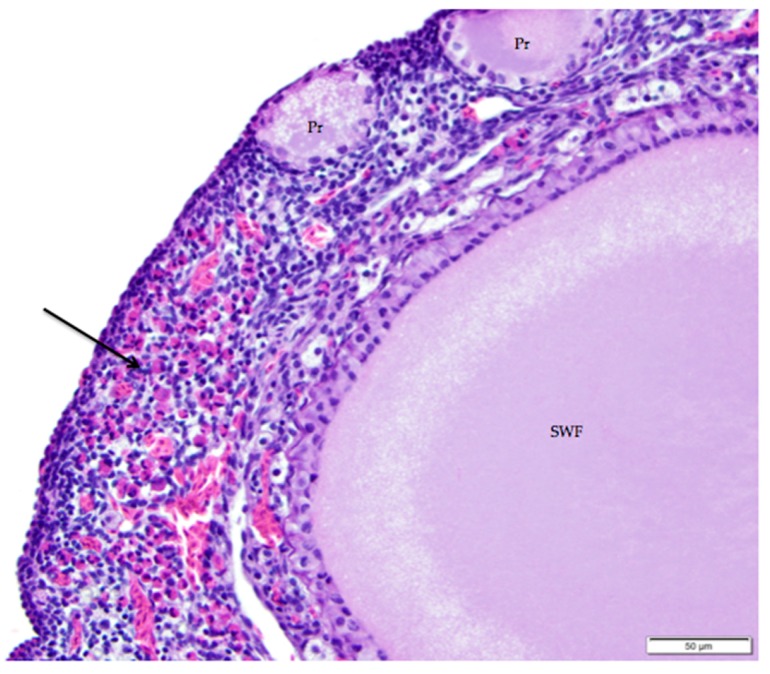
Infiltrates of heterophils (arrow) in the ovarian cortex are associated with atresia of small follicles. Primordial (Pr) follicles and a small white (SWF) follicle are labeled. H & E. 400×.

We propose that it is possible to distinguish post-ovulatory follicular regression from atresia of small follicles in normal ovaries of laying hens. Regressing post-ovulatory follicles are accompanied by characteristic changes in the vascular and connective tissue surrounding the collapsed follicle (Figure 8). These changes are absent in atresia of small follicles. In particular, we observed that infiltrates of heterophils are associated with atresia of small follicles (Figure 10). We commonly observed infiltrates of heterophils between existing follicles where small follicles would typically be present. Adjacent regions of cortex lacking the infiltrates of heterophils had expected numbers of small follicles. The heterophil infiltrates do not appear to be accompanied by histological evidence of acute inflammatory tissue damage or infection.

### 3.5. Infundibulum and Magnum of the Proximal Oviduct

**Figure 11:**
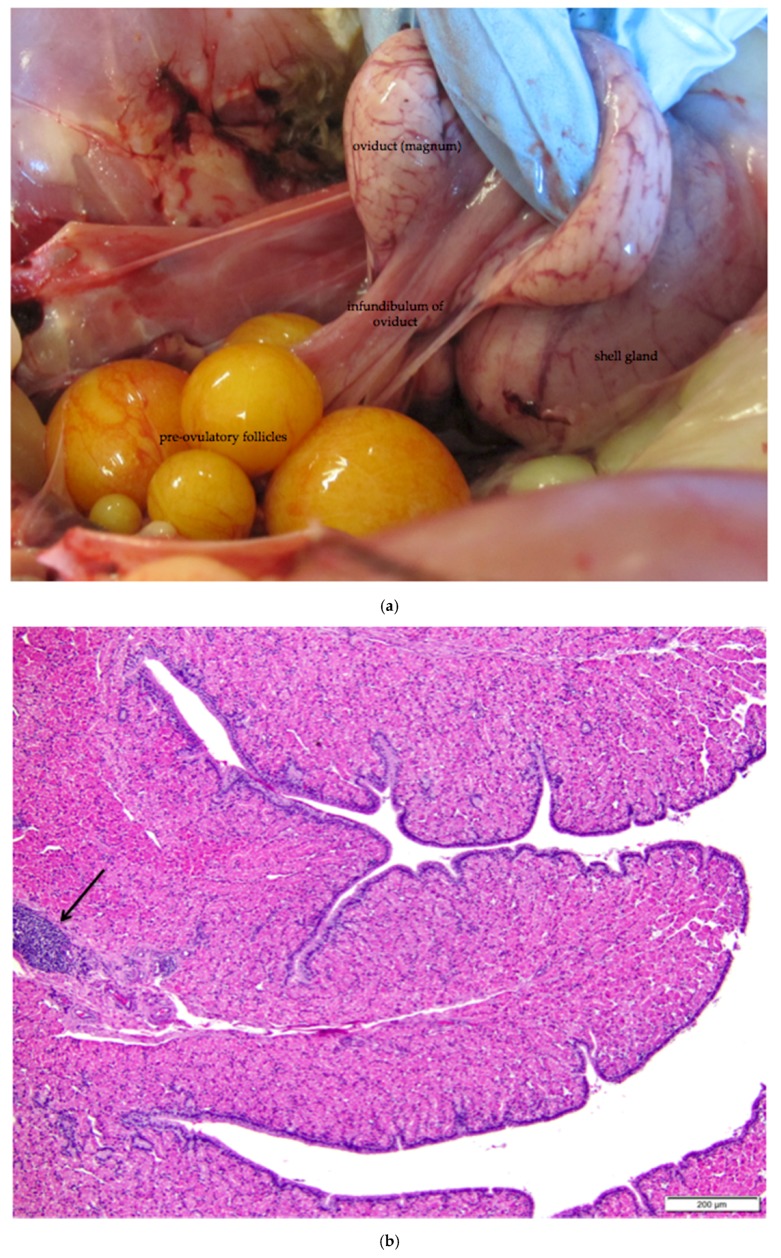

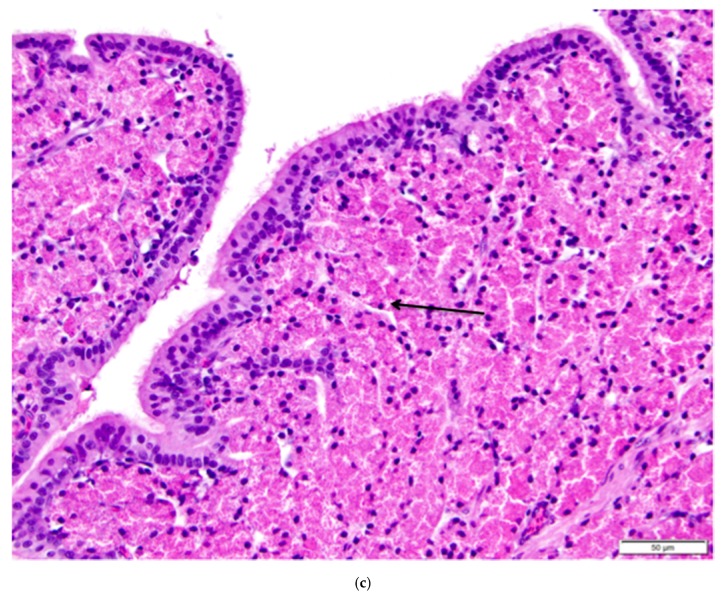
(**a**) Photograph of an in situ reproductive tract of a laying hen showing the relationship between the ovary with several large yellow pre-ovulatory follicles, the infundibulum of the oviduct, and the magnum of the oviduct; (**b**) The mucosa of the funnel-shaped infundibulum is folded into radial ridges that open out towards the ovary and spiral in towards the neck of the infundibulum. Lymphoid tissue is present in the mucosa and submucosa (arrow). H & E. 100×; **(c)** Cells lining tubular glands in the mucosal layer are filled with eosinophilic granules. The arrow indicates the nucleus of one of these cells. The surface epithelium of the infundibular ridges is comprised of ciliated columnar cells with large, oval nuclei. H & E. 400×.

The oviduct of the reproductively active laying hen has three primary functions: collection of the ovum from the ovary; deposition of albumen, membranes, and shell around the ovum; and transport of the developing egg to the cloaca. The oviduct is also the site of fertilization of the ova. In this paper, we focus on the histological characteristics of the infundibulum and the magnum of the oviduct (Figure 1 and Figure 11a). The mucosal layers of the oviduct are deeply and complexly folded and the characteristics of the epithelium in each section are related to the particular function of that section.

The infundibulum is a funnel-shaped structure lined with radial ridges of mucosa that spiral out from the neck and open towards the ovary. There are smaller secondary folds on the ridges. The ridges are comprised primarily of tubular glands in the mucosal layer. The glands are lined with cells containing eosinophilic granules, little discernible cytoplasm, and small, dense nuclei at the base of each cell (Figure 11b,c). Each mucosal ridge has a thin core of tunica submucosa (Figure 11b). Lymphoid tissue can frequently be observed in the mucosa and submucosa layers. The surface epithelium of the infundibulum is comprised of tall, ciliated, columnar cells with abundant cytoplasm (Figure 11c). Basophilic, oval nuclei, many with one or more nucleoli, are located near the center or at the base of the cells. Mucus-secreting goblet cells are interspersed with the ciliated cells towards the neck of the infundibulum.

**Figure 12:**
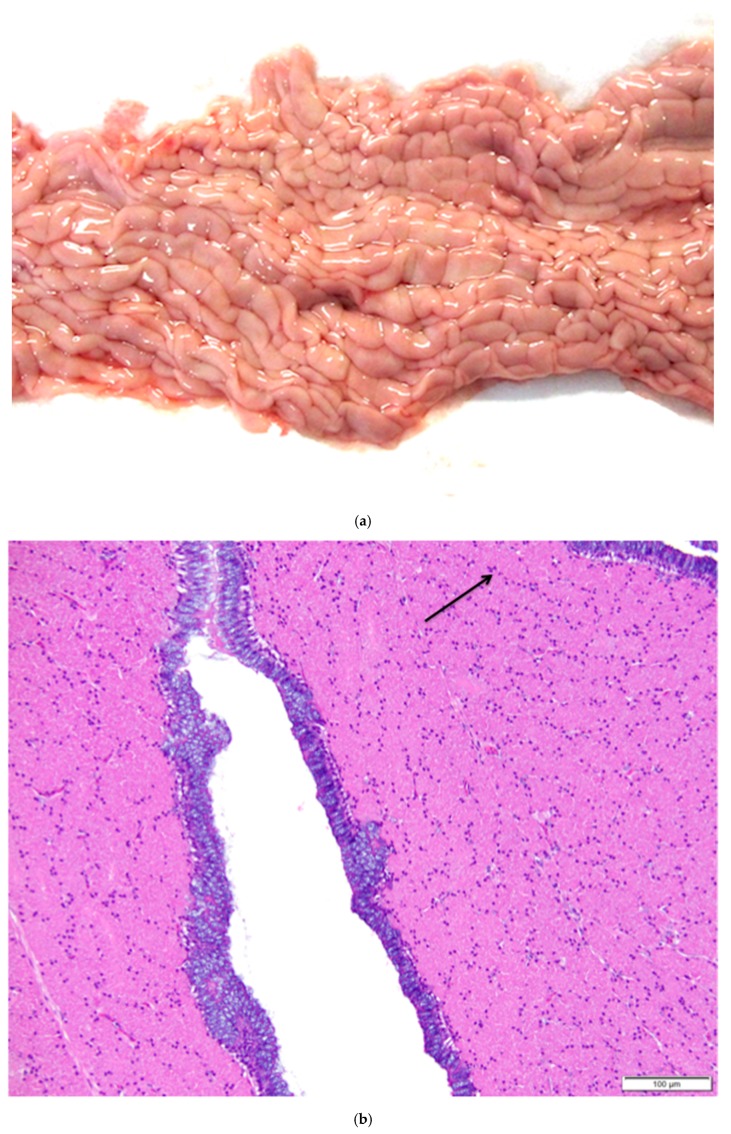
(**a**) Longitudinal section of the magnum of the oviduct in the laying hen showing thick, anastomosing folds of the mucosa. Width of the tissue section is about 3 cm; (**b**) Tubular glands are tightly packed into the mucosal folds. The glands are lined with cells filled with fine eosinophilic granules. The arrow indicates the nuclei in a group of these cells. The predominance of goblet cells over simple columnar epithelial cells in the surface epithelium indicates that this particular section was in a phase of active albumen secretion. H & E. 200×.

The magnum is the region in which albumen is secreted around the ovum. Both the muscularis and mucosal layers are significantly thicker than those in the infundibulum. The magnum is lined by anastomosing ridges and folds of mucosa (Figure 12a). Tubular glands in the mucosal layer are tightly packed into each fold. Cells lining the glands are densely packed with eosinophilic granules. As in the infundibulum, the folds have a thin core of submucosa. The surface epithelium is comprised of goblet cells and ciliated columnar cells. Goblet cells predominate during albumen secretion (Figure 12b) while the ciliated columnar cells predominate during a regenerative phase [27]. Different sections of the magnum can be in different phases. Lymphoid tissue is present in both mucosal and submucosal layers.

### 3.6. Rete Ovarii in the Laying Hen Ovary

In female animals, tissues of mesonephric origin, the rete ovarii, are comprised of three parts: the intraovarian rete, the connecting rete, and the extraovarian rete or epoöphoron. However, the particular components that are present vary with species and individual. We observed all three components of the rete ovarii in laying hen ovarian tissue samples collected in this study.

**Figure 13:**
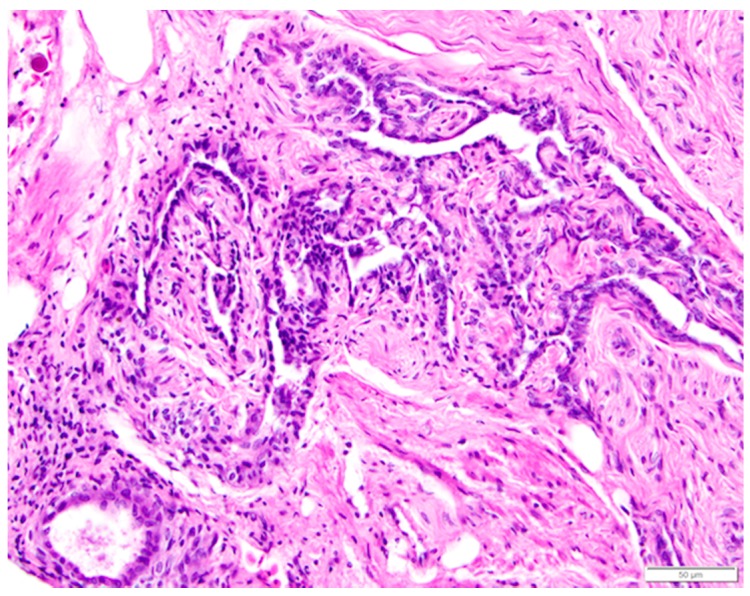
Basophilic, simple cuboidal epithelial cells lining intraovarian rete ovarii tubules are distinct from the eosinophilic connective and smooth muscle tissue of the ovarian medulla. A primordial follicle is in the lower left of the image. H & E. 400×.

The intraovarian and connecting rete structures in the laying hen are comprised of anastomosing tubules containing longitudinally aligned, flat-topped papillae that create a characteristic slit-like appearance (Figure 13). The papillae have collagenous cores extending from the ovarian medulla. The tubules are lined with basophilic, simple cuboidal epithelium containing small, dense, dark nuclei and small amounts of cytoplasm (Figure 13). The rete tubules are usually observed in the medulla in the region of the ovarian hilus (Figure 1b). The epithelial cells lining the connecting rete are ciliated while those of the intraovarian rete are not ciliated.

**Figure 14:**
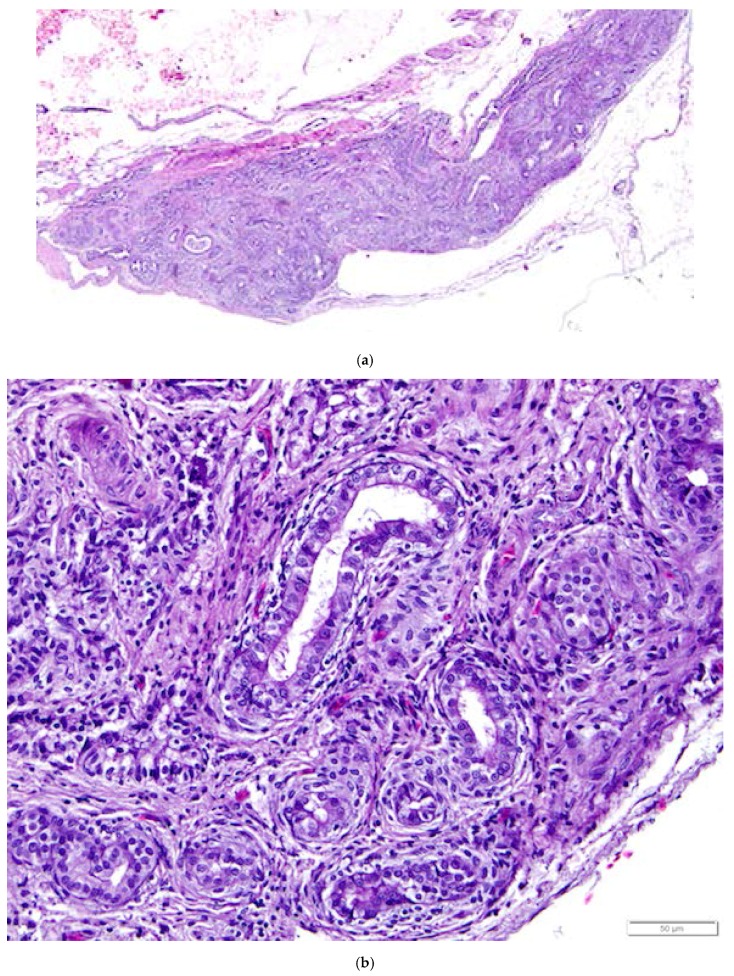
(**a**) Epoöphoron in the laying hen. This example is located entirely outside the ovary. H & E. 40×; (**b**) Round to oval, confluent gland-like structures with open lumens are lined with large, ciliated, simple cuboidal epithelial cells with pale to clear cytoplasm and large nuclei. The tubules are separated by connective and vascular tissue. H & E. 400×.

The gland-like tubules of the extraovarian rete or epoöphoron are distinctly different from the reticular channels of the intraovarian and connecting rete. In the laying hen, the epoöphoron can lie entirely outside the ovary in the mesosalpinx or mesovarium (Figure 1b and Figure 14a) or be integrated with ovarian medullary tissue in the hilar region. In laying hens, the epoöphoron is comprised of numerous, generally confluent, round to oval, gland-like tubules lined with tall, cuboidal to columnar epithelial cells with abundant basophilic cytoplasm (Figure 14b). Nucleoli are visible in most of the round to ovoid nuclei. Most of the euchromatic nuclei have a dark rim and relatively pale interior. Many of the epithelial cells lining the tubules are ciliated (Figure 14b). Most of the tubules have open lumens and some contain eosinophilic material. Fibroblasts and collagenous fibers surround each gland, and the abundant connective tissue is interspersed with blood vessels and numerous large, polyhedral cells with pale to clear cytoplasm and round to ovoid nuclei with distinct nucleoli. These cells are similar to stromal endocrine cells in the ovarian cortex. Ciliated connecting rete tubules can occasionally be observed in the epoöphoron, suggesting physical continuity between the structures.

### 3.7. Immunohistochemistry of the Laying Hen Ovary

**Figure 15:**
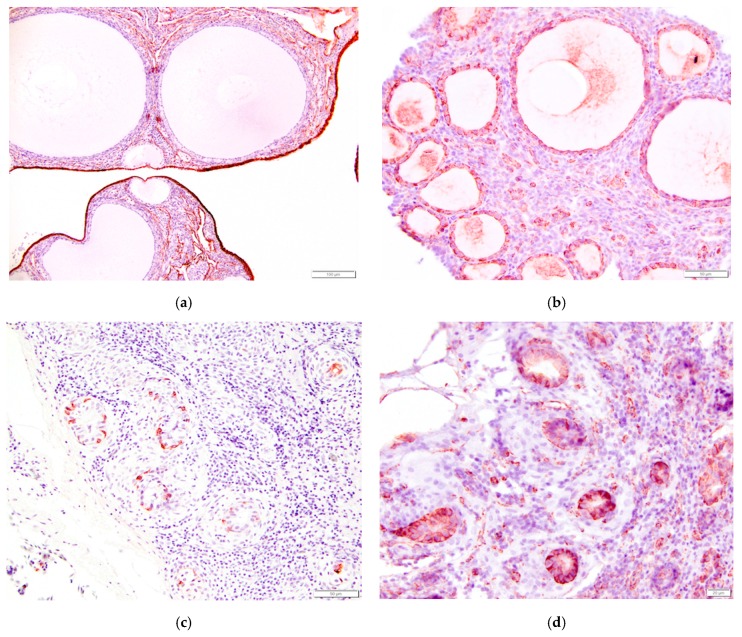
Immunohistochemical staining of tissues from the reproductive tract of the laying hen reveals a strong positive response to pancytokeratin in (**a**) ovarian surface epithelium (200×); (**b**) Granulosa cells of follicles had a strong positive response to vimentin (400×); Epithelial cells lining glands in the epoöphoron had a positive response to both (**c**) pancytokeratin (400×) and (**d**) vimentin (500×).

In order to further characterize the histology of normally functioning ovary of the laying hen, we performed immunohistochemical staining for pancytokeratin and vimentin. Similar to most mammals, ovarian surface epithelium stained strongly positive for pancytokeratin and negative for vimentin (Table 1, Figure 15a). Human ovarian epithelial cells have a similar response. Granulosa cells surrounding follicles in laying hen ovaries were negative for pancytokeratin and positive for vimentin (Figure 15b). The immunohistochemical response of remnant mesonephric tissues in the laying hen ovary is a new result of this study. Cells lining the intraovarian and connecting rete did not stain for pancytokeratin whereas cells lining the tubules of the epoöphoron were strongly positive for both pancytokeratin and vimentin (Figure 15c,d). Cells from epoöphoron tissues from women have a similar response.

## 4. Discussion

### 4.1. Ovarian Surface Epithelium

Epithelium of the ovary, oviduct, endometrium, and cervix in vertebrate animals is derived from coelomic mesothelium that overlies the embryonic gonadal ridge [17,32]. The differentiation of the ovarian surface epithelium from mesothelium occurs relatively late in embryological development of both mammals and birds. This differentiation is associated with the development of primordial germ cells into primary oocytes [17,24]. Ovarian surface epithelium retains pluripotential characteristics of both epithelium and mesenchyme [10,17,32,33,34]: (1) it has receptors for gonadotropins and hormones that are absent in peritoneal mesothelium; (2) it secretes proteases, growth factors, and cytokines such as IL-1 and IL-6; (3) it secretes proteins such as integrin and plasminogen activation inhibitor that are important for the repair activities that follow ovulation; and (4) it retains an ability to proliferate, also important in repair. At least two factors are thought to contribute to the potential for neoplastic changes in ovarian surface epithelial cells. The first is dysregulation of cell repair functions [32,35,36,37]. The second is accumulation of DNA damage in individual cells that is related to the incessant ovulation hypothesis for women and laying hens [8,10,19,35,36,38].

As we observed, simple squamous coelomic mesothelium smoothly transitions to the more typically cuboidal ovarian surface epithelium near the ovarian hilus, an observation consistent with other studies [33]. We observed variations in morphology of ovarian surface epithelium that may be related to the state of development of the underlying follicle. A similar change in morphology of the ovarian surface epithelium has also been observed in Japanese quail [39]. These changes are likely related to the steroidogenic and endocrine activity of the adjacent follicles and the stromal cells in the ovarian cortex that are in contact with ovarian surface epithelium [17].

### 4.2. Oviductal Origin for Some EOC Tumors

Epithelial cells in EOC tumors are not normal constituents of the ovary [20]. The tumors in women are classified into histological subtypes based on the similarity of the epithelial cells to other epithelial types [12,20]. Cells in serous tumors resemble ciliated epithelial cells of the proximal oviduct. Mucinous tumors contain cells that resemble epithelium of the endocervix or gastrointestinal mucosa. Endometrioid tumors are comprised of glands that resemble endometrial mucosa. A fourth histological subtype—called clear cell—is also similar to glands in endometrial mucosa. Although hens do not have endometrial mucosal glands, and their oviduct secretory functions differ considerably from those in women [12], they nonetheless develop tumors with the same histological subtypes [14].

There are several lines of evidence based on analysis of data from both women and laying hens that at least some EOC tumors may have an oviductal origin. In women, normal epithelial cells in the oviductal fimbriae and high-grade serous tumors in the OSE express p53 mutations [21]. In women [9,17] and laying hens [40,41], both Mullerian epithelia and EOC tumors express CA125 but normal OSE does not. Giles et al. [42] demonstrated that ovalbumin, the main protein secreted by the proximal oviduct in hens was expressed in EOC tumors in hens regardless of the presence of tumors in the oviduct. Trevino et al. [43] identified 273 genes that were differentially expressed between normal ovaries and cancerous ovaries of laying hens. Ten of the top 25 over-expressed genes were associated with oviduct functions. None of these genes were expressed at significant levels in normal ovarian surface epithelium. The expression of such genes as folate receptor and CA125 by EOC tumors may not truly be a case of over-expression. Instead, these genes are being normally expressed relative to the cell of origin (oviductal epithelium) and only abnormally expressed relative to the gene expression of OSE [41].

Solid tumors can be present in either or both the ovarian surface epithelium and oviduct in hens and women. Determination of which site is primary and which is metastatic may be difficult. It appears possible, even likely, that malignant cells from the oviduct can seed tumors in the OSE without forming solid tumors in the tissue of origin. Establishing a baseline for the histological appearance of the proximal oviduct (infundibulum and magnum) in laying hens that have not yet developed detectable tumors in either oviduct or ovarian surface epithelium may make identification of pre-neoplastic changes in these tissues more consistent.

### 4.3. Ovulation and the Follicular Hierarchy in Birds

The avian follicle hierarchy has been well documented [24,25,44,45,46,47]. In brief, primordial and primary follicles are less than 1 mm in diameter and are in a state of stasis and slow growth, respectively. They contain primary oocytes that are arrested in the first meiosis. These follicles are entirely enclosed within the ovarian cortex. Follicles that have developed past the primary stage bulge out from the surface of the avian ovary. Small white follicles that contain lipoproteins are defined to be 5 mm or less in diameter. Small yellow follicles, colored by an accumulation of lipids, can be up to 9 mm in diameter. Once follicles reach 8 or 9 mm in diameter, they begin a phase of rapid growth, including grossly visible increases in vascularization. This appears to coincide with the point at which both granulosa cells and thecal cells become steroidogenically competent [48], producing mostly progesterone, and androgens and estrogen, respectively.

During active reproductive periods, laying hens maintain five or six pre-ovulatory follicles with diameters increasing from 9 to 40 mm (Figure 1). Once follicles enter the pre-ovulatory hierarchy, they begin to express anti-apoptotic proteins [25]. After ovulation of the largest pre-ovulatory follicle (F1 follicle), the other pre-ovulatory follicles increase in size, with F2 becoming F1, F3 becoming F2, and so forth, while a single follicle is recruited from the small yellow pool to move into the follicular hierarchy. Johnson and Woods [25] and Johnson [46] discuss endocrine controls on follicle selection, a topic that is beyond the scope of the present paper.

The spacing between ovulations varies with species, and species-specific clutch size determines the total number of ovulations per breeding season in wild birds [24,44]. Laying hens have been genetically selected and are kept in artificial lighting and housing conditions such that seasonal controls on ovulation are overridden. After reaching sexual maturity around 18–20 weeks of age, laying hens ovulate approximately every 24 h. By the time that they are 24 months old, most hens have experienced several hundred ovulations, about the same number as a woman approaching menopause. The “incessant ovulation” hypothesis [10,19] helps explain why similar types of adenocarcinomas spontaneously develop in the ovarian surface epithelium of laying hens and women while ovarian tumors of other species occur more commonly in the stroma or germ cells [17].

In birds, ovulation of the largest pre-ovulatory follicle does not produce a corpus luteum, although there is considerable evidence that the regressing follicle acts as an endocrine structure for a few days [49]. For example, removal of the most recent post-ovulatory follicle before it has completed regression delays oviposition of the egg derived from that follicle [24]. The post-ovulatory follicle regresses in an orderly fashion via apoptosis [50] in which caspases and macrophages play an important role [25,37,51]. Our observation of macrophages containing hemosiderin within late-stage regressing follicles is consistent with this. Regression in the laying hen takes about 4–6 days [51].

Some sources mention scar tissue forming in association with regressed follicles (e.g., [23]). We observed an increase in connective tissue around and within large follicles during post-ovulatory regression. However, we found no histological evidence that fibrous or collagenous tissue persists in the form of a scar in the area of fully regressed follicles, an observation supported by Gilbert [24].

Atresia is an important process in avian ovaries. However, unlike in mammalian species, atresia of cohorts of follicles does not occur upon ovulation of the largest pre-ovulatory follicle in birds. Follicles in all states of development are maintained during the breeding season in the case of wild birds, or continuously in the case of commercial layers.

Because laying hens are kept in a continuous state of active reproduction, small follicles undergo continuous atresia with estimated rates that may be as high as 20% [25]. The most common form of atresia is in situ resorption [24,25], referred to as obliterative atresia by Barnes et al. [23]. Atresia occurs via an orderly apoptotic cascade similar to regression following ovulation [51] which is why the end result, small clusters of vacuolated cells, is the same in both processes. Atresia rarely occurs in large, post-ovulatory follicles under normal conditions of active reproduction in the laying hen.

Lymphocytes can be observed in and around regressing follicles and it is thought that they secrete cytokines that play a role in regulating this process [49,52]. Although it has long been noted that laying hens commonly have infiltrates of granulocytes in the ovarian cortex [23], the association between heterophils and atresia of small follicles has not previously been suggested. Avian heterophils are aggressive phagocytes [53,54,55]. We propose that heterophils are associated with atresia of small follicles in laying hens via phagocytosis.

Like mammalian neutrophils, heterophils are first responders in acute inflammation events, particularly those associated with infectious agents [53,54,55]. Although avian heterophils have a minor oxidative burst response, they can still cause tissue damage when they degranulate [53,54,56]. There is no evidence that the heterophil infiltrates described in this study are causing tissue damage associated with acute inflammation or are the result of an infectious event. For example, we did not observe hyperemia, abnormal follicles, or tissue necrosis that is characteristic of oophoritis, a condition that is generally associated with a bacterial infection (e.g., [23]).

### 4.4. Mesonephric Tissues in Laying Hen Ovaries

Tissues of mesonephric origin, such as the rete ovarii, are normal components of the reproductive tract of female animals of most, if not all, mammalian species [10,57]. These tissues are also present in adult laying hens [23,24,26] as well as in other avian species [23,24].

The histology of rete ovarii in laying hens has not been previously described in detail. Because these tissues are known to develop cysts, adenomas, and rarely, adenocarcinomas, in other species [58,59,60,61,62,63,64] and because the histology of the normal tissues is poorly documented, they can be mistaken for tumors in laying hen ovaries. Since the laying hen is an animal model for EOC, there is a need for a detailed description of the normal histological presentation of these tissues in the hen. As described in this study, rete ovarii in laying hens appear to be similar in histopathology, location, and structure to these tissues in other species.

The rete ovarii in female animals have the same embryonic mesodermal origin as mesonephric tissues in the male. This allows the rete ovarii components to be compared with their male homologues: the epoöphoron is the remnant of the epididymis, the connecting rete tubules are remnants of the ductus deferens, and intraovarian rete tubules are remnants of the rete testis [10,24,26].

The rete ovarii have an important role in initiating the first meiosis in oocytes. In many of the species studied, primordial germ cells are observed in mesonephric sex cords that are considered to be the precursors of the intraovarian rete tubules. Observations in juvenile mice and embryonic chicks suggests that meiosis only begins in germ cells that are in mesonephric cords that are also continuous with the ovarian surface epithelium [24,65]. The rete ovarii are also necessary for follicle formation. Removal of the rete ovarii before follicle formation results in the permanent absence of follicles in mice [66]. Rete tubule epithelium may be the source of some or all of the granulosa cells, although contributions from the ovarian surface epithelium cannot be ruled out [17,30,57,59,65,66].

The rete ovarii epithelium may also have a secretory function in adult animals. Eosinophilic material in the lumens of connecting rete tubules and the gland-like structure of the epoöphoron has been observed in numerous species [57], and we observed it in lumens of tubules of the epoöphoron in the laying hens examined in this study. In the adult female, the rete ovarii tubules are thought to have blind endings in the mesovarium, and in most species, the rete tubules commonly become increasingly cystic with age, consistent with persistent secretion from the rete epithelium. The cysts are usually benign and are often incidental findings.

### 4.5. Immunohistochemical Response of Ovarian Tissues to Pancytokeratin and Vimentin

Immunohistochemical staining of rete ovarii has been described for several species, including fetal and adult cow, rat, pig, mouse, chicken, and human [28,29,30,31] (Table 1). The reaction of selected cells in the female reproductive tract to pancytokeratin, an epithelial marker, and vimentin, an intermediate filament protein expressed by cells with a mesenchymal phenotype, varies with species and is not always a reliable indicator of embryonic origin [30].

Epithelium of the paramesonephric (Müllerian) ducts, which includes the ovary, oviduct, endometrium, and cervix in vertebrate animals, is derived from coelomic mesothelium that overlies the embryonic gonadal ridge [17,32]. Mesonephric (Wolffian) tubules develop from invaginations adjacent to the gonadal ridge and thus their epithelium has a similar embryonic mesodermal origin.

Ovarian surface epithelium has a positive reaction to pancytokeratin in humans, pigs, rats, mice, both fetal and adult cows, and laying hens. The response of the ovarian surface epithelium to vimentin is generally negative in the same species.

Follicles in the laying hen ovary are delineated by a single layer of granulosa cells that enclosed primordial germ cells as they migrated through mesonephric sex cords in the developing gonad to the ovarian cortex [24,25]. The embryonic origin of granulosa cells remains unresolved. It has been variously proposed that they are derived from ovarian surface epithelium (mesothelium), ovarian cortical stromal cells (mesenchyme), or from epithelium of mesonephric tissues (intraovarian rete ovarii) [30]. They may even have more than one embryonic origin. Granulosa cells stain for pancytokeratin in human and fetal cows, consistent with a mesothelial origin, but do not stain in the rat, pig, and adult cow. We did not observe granulosa cells staining for pancytokeratin in the laying hen ovary. Granulosa cells of human, pig, rat, mouse, cow stain for vimentin, consistent with a mesenchymal origin. We observed staining for vimentin in the laying hen ovary, consistent with results in [28]. Such mixed immunohistochemical responses cannot be used to definitively determine the embryonic origin of granulosa cells.

Rete tubule epithelium stains for pancytokeratin in humans and fetal cows but not in adult cows. In new findings of this study, we observed no staining for pancytokeratin in intraovarian rete epithelium of the laying hen, but a strong reaction in selected epithelial cells lining the gland-like structures of the epoöphoron. The response of rete tubule epithelium to vimentin has a mixed response in humans but is positive in the rat, pig, and fetal and adult cow. We observed that all remnant mesonephric epithelia in the laying hen stained strongly for vimentin. Interestingly, the human rete testis, the male homologue to intraovarian rete, does not stain for vimentin [30].

## 5. Conclusions

The laying hen is a robust model for epithelial ovarian cancer. We describe the microscopic histological characteristics of the normally functioning laying hen ovary and proximal oviduct to establish baselines from which pre-cancerous lesions can be more readily identified. The surface epithelium of the ovary can be simple squamous, cuboidal, or low columnar depending in part on the state of development of adjacent follicles. The transition of cuboidal ovarian surface epithelium to squamous mesothelium in the region of the ovarian hilus has not been previously described for the laying hen. Post-ovulatory follicular regression can be distinguished from atresia of small follicles by changes in vascularization and distribution of connective tissue with no evidence of persistent scar tissue. Localized infiltrates of heterophils are particularly associated with atresia of small follicles, a relationship not previously proposed for laying hens. We present a detailed description of the histology of the rete ovarii in the laying hen. We found that these remnant mesonephric tissues are similar to those in other species with respect to histology, structure, and location.

## Figures and Tables

**Table 1:** Immunohistochemical response of female reproductive tract epithelial cells to pancytokeratin and vimentin.

Tissue	Pancytokeratin	Vimentin
ovarian surface epithelium	+ laying hen (this study), [28] + human [29,30] + pig, rat, mouse [30] + mouse, fetal cow, adult cow [31]	− laying hen (this study), [28] − human [29] + human (periphery of the cells) [30] − pig [30] − rat, pig, fetal cow, adult cow [31]
granulosa cells	− laying hen (this study) + human [30] − pig, rat [30] + mouse, fetal cow [31] − adult cow [31]	+ laying hen (this study), [28] + human, particularly mature cells [30] + pig, rat, mouse [30] + fetal cow, adult cow [31]
rete ovarii epithelium	− laying hen (this study) + human [29,30] + fetal cow [31] − adult cow [31]	+ laying hen (this study) + human [29,30] − human rete testis [30] + rat, pig, fetal cow, adult cow [31]
epithelium lining tubules of epoöphoron	+ laying hen (this study) + human [29]	+ laying hen (this study) + human [29]
oviduct epithelium	+ laying hen (this study) + human [29]	+ human [29]
